# Retinal and choroidal efficacy of switching treatment to faricimab in recalcitrant neovascular age related macular degeneration

**DOI:** 10.1038/s41598-024-59632-0

**Published:** 2024-04-26

**Authors:** Franziska Eckardt, Anna Lorger, Michael Hafner, Julian Elias Klaas, Benedikt Schworm, Thomas Christian Kreutzer, Siegfried Georg Priglinger, Jakob Siedlecki

**Affiliations:** Department of Ophthalmology, LMU University Hospital, LMU Munich, LMU Augenklinik, Mathildenstraße 8, 80336 Munich, Germany

**Keywords:** Macular degeneration, Retinal diseases

## Abstract

Aim of this study was to evaluate the efficacy of switching treatment to faricimab in neovascular age-related macular degeneration (nAMD) from other anti-VEGF agents. Fifty-eight eyes of fifty-one patients with nAMD and a full upload series of four faricimab injections were included. Demographic data, multimodal imaging and treatment parameters were recorded. The primary outcome measures were changes in central subfield thickness (CST) and subfoveal choroidal thickness (SFCT). A subgroup analysis was performed for eyes with prior ranibizumab (R) or aflibercept (A) treatment. Mean injection intervals before and after switching were comparable (33.8 ± 11.2 vs. 29.3 ± 2.6 days; *p* = 0.08). Mean CST of 361.4 ± 108.1 µm prior to switching decreased significantly to 318.3 ± 97.7 µm (*p* < 0.01) after the third faricimab injection, regardless of prior anti-VEGF treatment (*p* < 0.01). Although SFCT slightly improved for the whole cohort from 165.8 ± 76.8 µm to 161.0 ± 82,8 µm (*p* = 0.029), subgroup analysis did not confirm this positive effect (subgroup R: *p* = 0.604; subgroup A: *p* = 0.306). In patients with a suboptimal response to aflibercept or ranibizumab in nAMD, farcimab can improve CST and slightly improve or maintain SFCT. Further prospective randomized trials are warranted.

## Introduction

Vascular endothelial growth factor (VEGF) plays an essential role in the pathogenesis of neovascular age-related macular degeneration (nAMD). The intravitreal application of anti-VEGF agents leads to suppression of endothelial cell proliferation, vascular permeability, and the development of choroidal or macular neovascularization (CNV/MNV)^1^. Thus, visual prognosis of nAMD has substantially improved in the past two decades^2^. Currently, ranibizumab (Lucentis®, Novartis), aflibercept (Eylea®, Bayer) and brolucizumab (Beovu®, Novartis) possess FDA and EMA approval, while bevacizumab (Avastin®, Roche Pharma) is used off-label^3^. MNV activity in nAMD is mostly defined as an increasing central macular thickness (CST), new macular fluid including intraretinal fluid (IRF), subretinal fluid (SRF), sub-RPE fluid (sub-RPEF) or pigment epithelium detachment (PED)^2,4,5^ . In daily clinical practice, patients with a poor response to anti-VEGF injections require close follow-up and monthly retreatment, resulting in a significant disease burden for patients and not inconsiderable cost^4,6^ .

In 2022, Faricimab (Vabysmo®, Roche/Genentech) was approved by the FDA and EMA as novel bispecific anti-VEGF/Angiopoietin 2 (Ang-2) inhibitor for the treatment of nAMD. As Ang-2 promotes vascular destabilization and induces an amplification of VEGF signaling through the Tie-2 pathway, Ang-2 markedly contributes to the pathological neovascular processes observed in nAMD^7^. Thus, an Ang-2 inhibitor has the potential to contribute to reduced vascular remodeling and vascular stabilization^1,8^. The safety and efficacy of faricimab was shown in several studies^9–12^. According to the phase 3 studies LUCERNE and TENAYA, faricimab might allow for more efficient disease management with an extension of the injection interval up to 16 weeks after a loading phase of 4 monthly doses^9^.

In addition to a possible reduction in therapeutic burden, new intravitreal agents for nAMD can also represent a good switching alternative for patients with a suboptimal treatment response to established substances. Aim of this retrospective real-world study therefore was to evaluate the efficacy of faricimab in patients with a poor response to prior nAMD treatment with ranibizumab and aflibercept, focusing on its effects on the retina and choroid.

## Methods

### Participants

For this retrospective study, the Smart Eye Database (SmEyeDat) of the Department of Ophthalmology, LMU University Hospital Munich, was screened for patients treated with faricimab for neovascular AMD between October 2022 and July 2023. Inclusion criteria were defined as (i) full loading phase of at least three faricimab injections; (ii) prior intravitreal therapy in our department; (iii) inadequate response to ranibizumab/aflibercept treatment, defined as persistence of intraretinal/subretinal-fluid in spite of monthly anti-VEGF therapy or the inability to increase injection intervals further than four to six weeks (fluid recurrence at seven weeks); (iv) absence of confounding factors, e.g. intraocular infection or uveitis. Institutional review board approval was obtained from the Ethics committee of the Faculty of Medicine, LMU Munich (study identifier 23–0129) and the study adhered to the tenets of the Declaration of Helsinki. All patients provided written informed consent. Epidemiological data was obtained from each patient, including age, gender, date of first nAMD diagnosis, number of previous intravitreal injections therapies, date of switch to faricimab. During therapy, every patient underwent a clinical assessment including best corrected visual acuity (BCVA), slit lamp examination, fundoscopy and optical coherence tomography (SD-OCT) at every presentation.

### Multimodal imaging

Multimodal imaging was performed as needed and included SD-OCT and near infrared (NIR) scanning using the Spectralis HRA + OCT, Heidelberg Engineering system at each visit. At first diagnosis, fluorescein angiography was performed prior to treatment initiation. Automated CST measurements were obtained from the Heidelberg Eye Explorer (Version 1.10.12.0) after segmentation was manually corrected, if needed. Subfoveal choroidal thickness (SFCT) was measured directly under the fovea in the 1:1 µm display mode from the outer portion of the retinal pigment epithelium to the sclerochoroidal interface. If the chorioscleral border could not be visualized due to a thick choroid, the EDI mode (enhanced depth imaging) was additionally switched on. OCT data was collected from the the date of therapy initiation for nAMD (T1), the last three visits with intravitreal injections before switching to faricimab (T2-T4) and the first three faricimab intravitreal injections of loading phase (T5-T7).

### Anti-VEGF treatment

Prior injection therapy included aflibercept (subgroup A) or ranibizumab (subgroup R). All patients received a faricimab loading of three injections every 30 days ± 7 days, after which the interval was extended based on the discretion of the doctor. Each injection contained 6 mg faricimab. MNV activity was defined as new macular fluid including IRF and SRF, PED or increasing CST.

### Data analysis and statistics

Data management was done using Microsoft Excel Version 16.72 for Mac. Statistical analysis was performed in IBM SPSS® Statistics 28 (IBM Germany GmbH). Significance level was set at *p* < 0.05. Shapiro–Wilk test showed no normal distribution. Friedman test was used to analyze CST during the treatment period. Post hoc analysis was then applied for the pairwise comparison of the values. The Cohen´s effect size of the Friedman test was calculated using the formula r = (z/√n), where r < 0.3 corresponds to a weak effect, 0.3—0.5 to a medium effect, and > 0.5 to a strong effect.

### Visual acuity measurements:

Visual acuity (VA) was documented in decimal VA and then converted to logarithm of the minimum angle resolution (LogMAR) units for analysis. Visual acuity was measuret throughout faricimab loading phase (T4-T7) or a small number of patients, the visual acuity values were missing at previous time points, particularly at initial diagnosis. The electronic chart had not yet been established for all patients at that time, so no visual acuity values were recorded electronically.

## Results

### Baseline demographics

In total, 58 eyes of 51 patients were included in our analysis. Baseline demographics are summarized in Table 1. Average age at nAMD diagnosis was 75.2 ± 7.1 years, gender ratio was 25 male and 33 female. On average, patients received 37.5 ± 25.9 anti-VEGF injections prior to switching, consisting of 13.5 ± 14.7 ranibizumab and 24.0 ± 21.6 aflibercept injections on average. Average period from initial treatment to the point of switching to faricimab was 4.2 years. All faricimab injections were performed every 30 days ± 7 days. Mean injection intervals before and after switching were comparable (33.8 ± 11.2 vs. 29.3 ± 2.6 days; *p* = 0.08). There were no severe ocular complications recorded during this follow up period (no cases of intraocular inflammation, retinal detachment, severe spikes in IOP, intraocular hemorrhages or tears of the retinal pigment epithelium).

**Table 1:** Baseline demographics, including age, gender, CNV type and prior intravitreal injection therapy.

**Number of patients**	51
**Number of eyes**	58
Right	27
Left	31
**Mean age (years)**	75.2 ± 7.1
**Gender**	
**Male**	25
**Female**	33
**MNV type**	
**1**	25 (43.1%)
**2**	20 (34.5%)
**3**	13 (22.4%)
**Mean prior anti-VEGF injections**	
**Total (n)**	37.5 ± 25.9
**Total RBZ**	13.5 ± 14.7
**Total AFL**	24.0 ± 21.6
**Mean injections/year**	9.4 ± 2.5
**Last injection**	
Ranibizumab	14 (24.1%)
Aflibercept	44 (75.9%)
**Injection interval before switch (months)**	1.33 ± 0.85

### Fluid, CMT and SFCT dynamics

**Figure 1:**
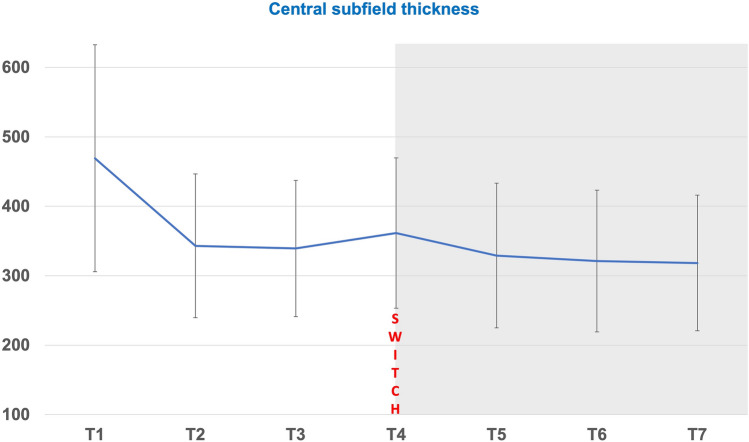
Central subfield thickness (CST) progress before (T2-T4) and after (T5-T7) switch to faricimab (T4).

**Figure 2:**
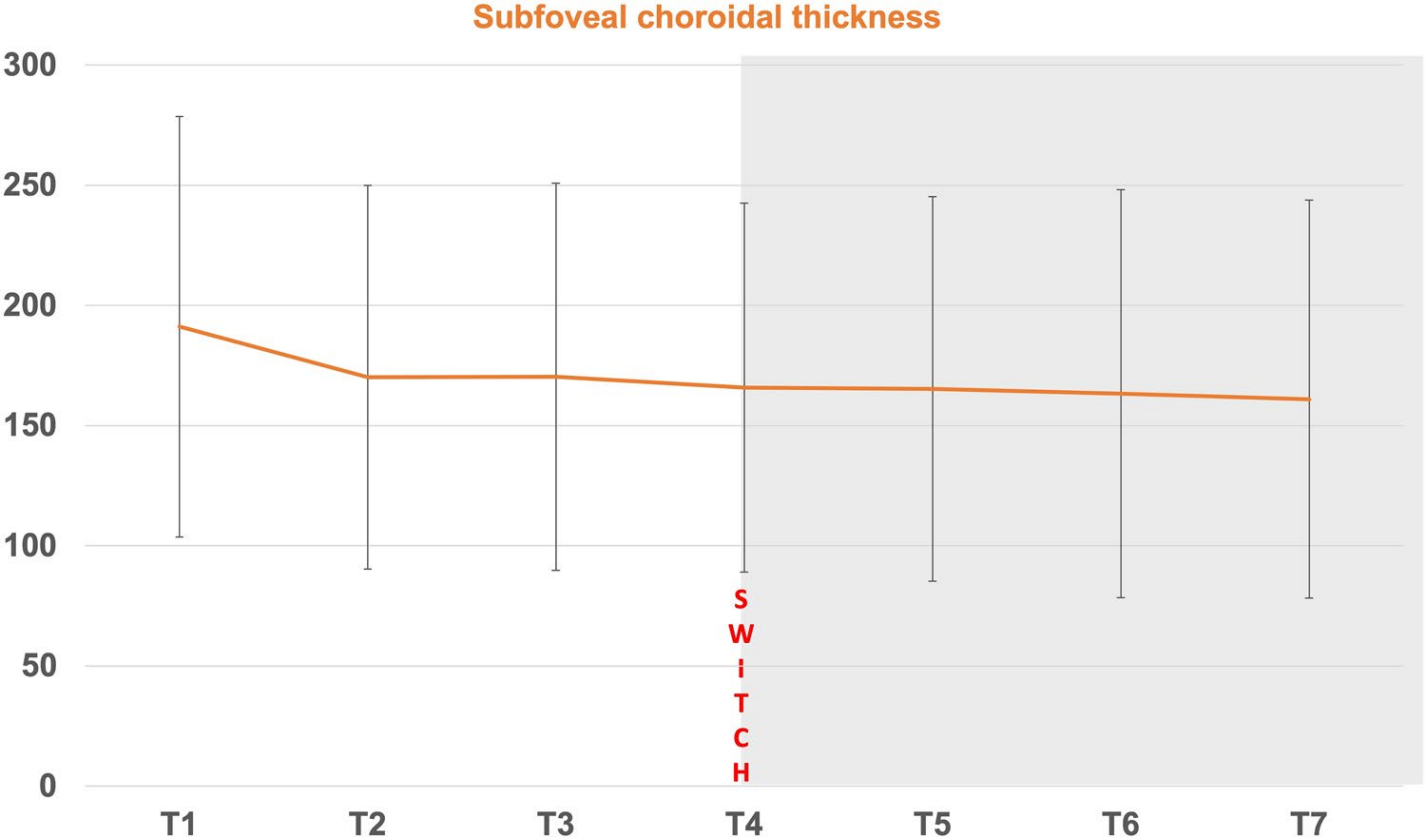
Subfoveal choroidal thickness (SFCT) progress before (T2-T4) and after (T5-T7) switch to faricimab (T4).

At baseline, all eyes showed MNV activity with IRF/SRF or PED. OCT at initial diagnosis showed PED in 57 eyes (98.3%), SRF was detected in all eyes and 20 eyes (34.5%) had additional IRF. CST measurements at each visit are shown in Table 2. Friedman Test showed a significant difference between measurements during T1-T7 (*p* < 0.01) (Table 3). Reduction of CST during the study period is shown in Figs. 1 and 2. CST improved from T1 to T4, however not significantly (*p* = 0.102). Between T2 and T4, CST remained similar (*p* = 0.184). Further improvement was noted from T4 to T7 (after three faricimab injections). Paired comparison showed a significant difference between start (T4) and end (T7) of faricimab loading dose (*p* < 0.01, r = 0.4). After switching, an improvement in CST was noted in 91.4% (53 eyes). In five eyes (8.6%), an increase in CST compared to OCT measurement at the baseline visit was noted. 19 eyes (32.8%) had remaining IRF, 4 eyes SRF ( 6.9%) and 2 eyes IRF and SEF (3.4). In total, 33 out of 58 eyes (56.0%) had a completely dry macula after the third faricimab injection. Remaining PED at the end of loading dose was detected in 26 eyes (44.8%).

**Table 2:** Central subfield thickness (CST) measurements during study period (T1-T7).

Time points	Mean CST (µm) all eyes (n = 58)	Mean CST (µm) ranibizumab subgroup (n = 14)	Mean CST (µm) aflibercepts subgroup (n = 44)
T1	469.22 ± 163.27	468.79 ± 180.57	469.36 ± 159.62
T2	343.03 ± 103..60	389.79 ± 139.30	328.16 ± 86.16
T3	339.29 ± 98.06	375.57 ± 131.83	327.75 ± 83.23
T4	361.45 ± 108.14	412.00 ± 140.76	345.36 ± 91.73
T5	329.07 ± 104.32	365.71 ± 140.09	317.41 ± 88.97
T6	321.16 ± 101.99	359.36 ± 119.38	309.00 ± 94.10
T7	318.29 ± 97.70	354.07 ± 125.63	306.91 ± 85.62

**Table 3:** Central subfield thickness in pairwise comparison.

Time points	Interpretation	Pairwise comparison, *p*- value
T1–T2	initial diagnosis—3 months pre-switch	** < 0.001**
T1–T4	initial diagnosis—switch	0.102
T2–T4	3 Months pre-switch—switch	0.184
T4–T5	after first faricimab injection	** < 0.001**
T4–T6	after second faricimab injection	**0.002**
T4–T7	after third faricimab injection	** < 0.001**
T5–T7	after first vs. third faricimab injection	0.664

Bold**:** significant *p*-values.

**Figure 3:**
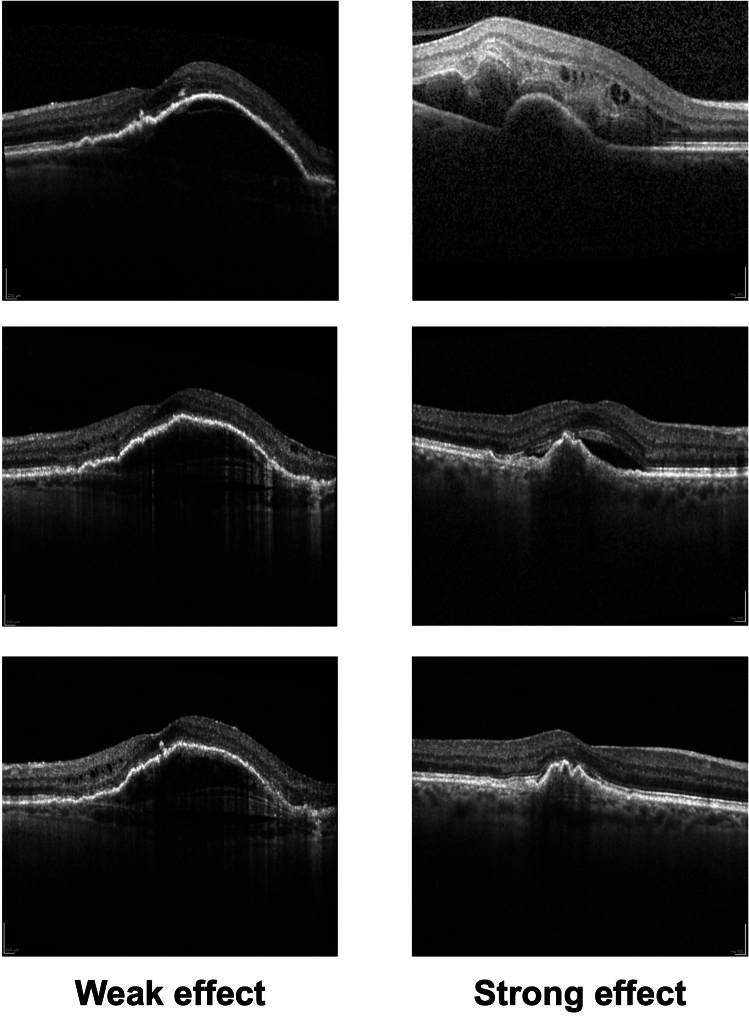
Examples of faricimab treatment effect. Top: Treatment naïve; Middle: T4 before switch to faricimab; Bottom: T7 after third faricimab injection.

SFCT measurements at each visit are shown in Tables 4 and 5. SFCT also decreased significantly, although the effect power was low (*p* = 0.029, r = 0.2). Figure 3 shows examples of faricimab treatment effect in OCT.

**Table 4:** Subfoveal choroidal thickness (SFCT) measurements during study period (T1-T7).

Time points	Mean SFCT (µm) all eyes ( n = 58)	Mean SFCT (µm) ranibizumab subgroup (n = 14)	Mean SFCT (µm) aflibercepts subgroup (n = 44)
T1	192.14 ± 87.42	207.64 ± 89.29	187.20 ± 87.28
T2	170.90 ± 79.83	180.64 ± 51.15	166.73 ± 87.24
T3	170.29 ± 80.59	186.29 ± 55.84	165.20 ± 86.94
T4	165.76 ± 76.81	178.93 ± 49.97	161.57 ± 83.61
T5	165.26 ± 80.05	177.21 ± 50.87	161.45 ± 87.47
T6	162.28 ± 84.79	167.50 ± 45.98	161.93 ± 94.26
T7	160.98 ± 82.75	171.79 ± 61.21	157.55 ± 88.85

**Table 5:** Subfoveal choroidal thickness in pairwise comparison.

Time points	Interpretation	Pairwise comparison, *p*- value
T1–T2	Initial diagnosis—3 months pre-switch	**0.005**
T1–T4	Initial diagnosis—switch	**0.030**
T2–T4	3 Months pre-switch—switch	1.0
T4–T5	After first faricimab injection	1.0
T4–T6	After second faricimab injection	0.283
T4–T7	After third faricimab injection	**0.029**
T5–T7	After first versus third faricimab injection	**0.038**

Bold: significant *p*-values.

In a subgroup analysis, CST and SFCT were examined during the treatment period in dependence on the prior drug ranibizumab or aflibercept just before the switch to faricimab. In total, 14 patients received ranibizumab (subgroup R), 44 patients received aflibercept (subgroup A). CST decreased significantly in both groups (subgroup R: *p* = 0.014 r: 0.5; subgroup A: *p* < 0.01, r: 0.2). Although SFCT slightly improved for the whole cohort, subgroup analysis did not confirm this positive effect (subgroup R: *p* = 0.604; subgroup A: *p* = 0.306).

### Visual acuity measurements

Visual acuity did not change significantly during loading phase with farcimab (*p* = 1,0). Mean visual acuity measurements are shown in Table 6 and pairwise comparison between time point is demonstrated in Table 7.

**Table 6:** Visual acuity (VA) measurements during study period (T1-T7).

Time points	Mean VA (LogMAR) all eyes	Mean VA (LogMAR) ranibizumab subgroup	Mean VA (LogMAR) aflibercepts subgroup
T1	0.50 ± 0.39 n = 58/58	0.43 ± 0.32 n = 12/14	0.53 ± 0.41 n = 40/44
T2	0.35 ± 0.33 n = 52/58	0.29 ± 0.25 n = 13/14	0.37 ± 0.35 n = 44/44
T3	0.53 ± 0.27 n = 57/58	0.62 ± 0.29 n = 13/14	0.51 ± 0.26 n = 44/44
T4	0.36 ± 0.32 n = 58/58	0.37 ± 0.35 n = 14/14	0.36 ± 0.31 n = 44/44
T5	0.38 ± 0.37 n = 58/58	0.49 ± 0.47 n = 14/14	0.35 ± 0.33 n = 44/44
T6	0.54 ± 0.27 n = 58/58	0.5 ± 0.25 n = 14/14	0.56 ± 0.27 n = 44/44
T7	0.53 ± 0.26 n = 58/58	0.41 ± 0.35 n = 14/14	0.36 ± 0.31 n = 44/44

**Table 7:** Visual acuity (VA) measurements in pairwise comparison.

Time points	Interpretation	Pairwise comparison, *p*- value
T1–T2	Initial diagnosis—3 months pre-switch	0.082
T1–T4	Initial diagnosis—switch	0.584
T2–T4	3 Months pre-switch—switch	1.0
T4–T5	After first faricimab injection	1.0
T4–T6	After second faricimab injection	**0.026**
T4–T7	After third faricimab injection	1.0
T5–T7	After first vs. third faricimab injection	1.0

Bold: significant *p*-values.

## Discussion

The clinical introduction of faricimab as a novel treatment for nAMD is of particular interest in ophthalmology^13^. This study confirms that patients inadequately managed with ranibizumab and aflibercept for nAMD can benefit from switching treatment to faricimab to improve CST and maintain, or even slightly improve SFCT.

In this study, an inadequate response to anti-VEGF treatment was defined as persistent fluid in spite of 4-weekly dosing, or the inability to extend treat & extend intervals beyond 4 or 6 weeks. In real life studies with ranibizumab and aflibercept, continuous treatment intervals of 4 weeks were shown to be needed in 19–27% of eyes^14,15^, indicating a large population of patients possibly benefitting from treatment switches to newer, longer-lasting anti-VEGF agents.

To this end, novel anti-VEGF molecules as well as bispecific approaches have been investigated. Faricimab as a bispecific antibody against VEGF-A and the Ang2/Tie-2 pathway raised promising expectations in preclinical evidence in terms of vascular stability and reducing leakage^16^ which were confirmed in large phase III clinical trials^7,9^.

To our knowledge, only a few trials have been conducted to investigate the efficacy of faricimab in a real-world setting since its approval^13,17–24^. These few published studies are mainly related to therapy-naïve patients, and there is little data on the outcome of faricimab therapy after pretreatment with other intravitreal drugs. Aim of the present study was to evaluate the short term response after switching to faricimab treatment following prior anti-VEGF treatment.

In the current study we focused on the morphological outcomes in OCT during loading phase of faricimab. OCT allows for a fast, accurate and quantitative assessment of nAMD and has established itself as an effective diagnostic standard in AMD^5,25^. CST is one of the main CNV activity markers, it provides a good readily comparable reference point^5^. Beside CST being an OCT progression marker, SFCT should also be of interest. Growth of choroidal vessels is affected by the retinal pigment epithelium mediated by VEGF^16,26,27^. There are controversial findings in literature, however VEGF inhibition can lead to a SFCT thinning by inducing vasoconstriction and reduction of choriocapillaris endothelial cell fenestrations. Additionally SFCT thinning could also be secondary to suppression of the CNV activity^28^. Not only VEGF receptors are expressed in the choroid, furthermore Ang1/Tie2 receptors, maintain the choroidal vessels^29^. As faricimab affects both receptors, its impact could be greater than anti-VEGF inhibitors.

Although all eyes in the present study had a long history of pretreatment, having received on average 38.4 ± 26.7 anti-VEGF injections before starting faricimab, the clinical response was only limited. In spite of the heavy pretreatment, faricimab nevertheless allowed for improvements in CST. Additionally, limited effects on SFCT were observed. SFCT, representing the perfusion origin of MNV, has been shown to influence anti-VEGF outcomes in nAMD eyes, especially those with a thick-choroid phenotype, e.g. PCV^30^. As this study presents the short-term effects after the loading phase, further studies assessing the possibility of treatment interval extension based on these.

Tenaya and Lucerne as phase 3 clinical studies compared faricimab to aflibercept in treatment naïve eyes. Faricimab provided equal functional and morphological outcomes with an interval of up to sixteen weeks^9^. This phase 3 studies were conducted with treatment naïve eyes, so a greater improvement in VA and CST in comparison with pre-treated eyes is expected^13^. Despite these results raising promising expectations, the effect of faricimab in clinical practice now needs to be verified in treatment naïve and pretreated patients. In this context, our data present real-world outcomes of treatment switch in pretreated eyes. Our findings correlate well with other studies on the subject. Stanga et al. evaluated VA and CST in 11 eyes early on after approval. Follow up time was only four weeks in this early published study. Both VA and CST improved^17^. Although those first and early results seemed to be promising, follow up time was short and cohort size small. Two Japanese studies of 2023 showed a decrease in central foveal and choroidal thickness, as well as an increase in VA within the first 3 month of faricimab loading in treatment naïve patients^18,29^. Overall, the morphological outcomes after faricimab were comparable to those after aflibercept treatment^23^. Even in treatment resistant AMD, faricimab can lead to an improved CST and VA even in treatment resistant AMD^13,31^. Kichi et al. found a decrease in CST without an improvement in VA, however treatment intervals could be prolongated^32^. Consistent with existing studies, we could verify these results in the documented CST decrease.

In line with recent studies investigating the effect of faricimab in pre-treated eyes, we did not find any significant improvement in visual acuity within the loading phase^19–22^. As shown in Table 6, the visual acuity values are highly variable. Visual acuity measured at a single measurement point in everyday clinical practice is highly dependent on the examiner, general condition and cohabitation. Therefore, the main criterion, especially in such a short period of time as the loading phase, are the image morphological changes. Interestingly, improvements were readily seen after the first faricimab injection. However, subsequent injections maintained this effect but did not significantly improve it. The effect power of 0.4 corresponds to a medium sized effect. Among patients receiving ranibizumab therapy immediately before the switch, the effect was greater compared to patients previously receiving aflibercept. On the other hand, not every eye showed a favorable response to faricimab. As shown in Fig. 2, eyes with a weak faricimab response do exist and reswitching to prior treatment should be considered.

There are certain limitations to this study, including its small sample size and short-follow up. Longer and larger trials, ideally designed as randomized cross-over trials, are warranted. Moreover, patients included in this study had undergone a relatively higher number of injections before switching to faricimab. Therefore, the extensive duration of prior treatment could mask an even better anatomical response in naïve or short-term treated eyes. Additionally, real world usability of new pharmaceutical agents does not only rely on efficacy, but also on safety. Our study was not powered to detect rare events like intraocular inflammation, however, our study does not add new adverse events to the published data.

In conclusion, our data support the efficacy of switching treatment in eyes with nAMD not adequately responding to older anti-VEGF agents. Future studies should observe not only the ability to extend treatment intervals, but also the long-term course both functionally and morphologically.

## Data Availability

The datasets generated during and analysed during the current study are available from the corresponding author on reasonable request.
